# An observational study of nutrition and physical activity behaviours, knowledge, and advice in pregnancy

**DOI:** 10.1186/1471-2393-13-115

**Published:** 2013-05-20

**Authors:** Susan J de Jersey, Jan M Nicholson, Leonie K Callaway, Lynne A Daniels

**Affiliations:** 1 Department of Nutrition and Dietetics, Royal Brisbane and Women’s Hospital, Herston, QLD, 4029, Australia; 2 School of Exercise and Nutrition Sciences, Institute of Health and Biomedical Innovation, Queensland University of Technology, Kelvin Grove, QLD, 4059, Australia; 3 Parenting Research Centre, East Melbourne, VIC, 3002, Australia; 4 Centre for Learning Innovation, Queensland University of Technology, Kelvin Grove, QLD, 4059, Australia; 5 Royal Brisbane and Women’s Hospital Clinical School, School of Medicine, University of Queensland, Herston, QLD, 4029, Australia; 6 Department of Internal Medicine, Royal Brisbane and Women’s Hospital, Herston, QLD, 4029, Australia

**Keywords:** Pregnancy, Nutrition, Dietary intake, Physical activity, Knowledge, Advice

## Abstract

**Background:**

Maternal obesity, excess weight gain and lifestyle behaviours during pregnancy have been associated with future overweight and other adverse health outcomes for mothers and babies. This study compared the nutrition and physical activity behaviours of Australian healthy (BMI ≤ 25 k/m^2^) and overweight (BMI ≥ 25 kg/m^2^) pregnant women and described their knowledge and receipt of health professional advice early in pregnancy.

**Methods:**

Pregnant women (n=58) aged 29±5 (mean±s.d.) years were recruited at 16±2 weeks gestation from an Australian metropolitan hospital. Height and weight were measured using standard procedures and women completed a self administered semi-quantitative survey.

**Results:**

Healthy and overweight women had very similar levels of knowledge, behaviour and levels of advice provided except where specifically mentioned. Only 8% and 36% of participants knew the correct recommended daily number of fruit and vegetable serves respectively. Four percent of participants ate the recommended 5 serves/day of vegetables. Overweight women were less likely than healthy weight women to achieve the recommended fruit intake (4% vs. 8%, p=0.05), and more likely to consume soft drinks or cordial (55% vs 43%, p=0.005) and take away foods (37% vs. 25%, p=0.002) once a week or more. Less than half of all women achieved sufficient physical activity. Despite 80% of women saying they would have liked education about nutrition, physical activity and weight gain, particularly at the beginning of pregnancy, less than 50% were given appropriate advice regarding healthy eating and physical activity.

**Conclusion:**

Healthy pregnancy behaviour recommendations were not being met, with overweight women less likely to meet some of the recommendations. Knowledge of dietary recommendations was poor and health care professional advice was limited. There are opportunities to improve the health care practices and education pregnant women received to improve knowledge and behaviours. Pregnant women appear to want this.

## Background

At least one third of pregnant women are overweight around the time of conception [1,2]. More than one third of women gain too much weight during pregnancy [3]. Pre-pregnancy weight status and excess pregnancy weight gain are important risk factors for future overweight in both mothers and babies [4]. Nutrition and physical activity behaviours are key modifiable factors associated with weight related outcomes in pregnancy. An understanding of these behaviours, including their variability according to pre-pregnancy weight status, knowledge of key recommendations and the provision of advice from health care providers is important for developing interventions to influence nutrition and physical activity.

Few pregnant women in Australia meet recommended food and nutrient intake or levels of physical activity [5]. The average intake of vegetables has been reported at approximately 2–2 ½ serves per day [6,7] when five are recommended, and only 9- 13% of women met the recommendations of four serves of fruit per day [6,7]. In the Australian Longitudinal Study on Women’s Health, none of the participating pregnant woman (n=606) met recommendations for all food groups [8]. Similarly, an Australian study (n=262) reported only a third of pregnant women met the American College of Obstetrics and Gynaecology (ACOG) [9] recommendation of 30 minutes or more of moderate exercise on most if not all days of the week [6].

The reasons why few women achieve recommended nutrition and physical activity guidelines during pregnancy are unclear. Knowledge and support are likely to be important. While knowledge is considered an essential precursor for behaviour change processes [10], it may not be sufficient for change. Women’s pre-pregnancy lifestyle behaviour and prior success with weight control may also be influential and contribute either positively or negatively to behaviour change. Health care professionals who have repeated contact with pregnant women potentially play a central role in providing education and supporting women’s intentions and efforts towards achieving healthy behaviour [11,12].

To date, little is known about the relationship between women’s nutrition and physical activity knowledge and behaviour in pregnancy. We also have little information about the advice received from health professionals. Moreover, it is unknown whether these factors differ for women who commence their pregnancy at a healthy weight compared to overweight. Understanding these factors is important to ensure appropriately targeted advice to support the achievement of healthy behaviour during pregnancy. This study aimed to describe the nutrition and physical activity behaviour and knowledge of healthy and overweight women (according to their pre-pregnancy BMI) in early pregnancy, and their receipt of health professional advice about nutrition and physical activity.

## Methods

### Study design and participants

The New Beginnings Healthy Mothers and Babies Study was a prospective observational study examining nutrition and physical activity behaviours and behavioural influences during pregnancy and the early post partum period. Participants were recruited through a public tertiary teaching hospital that provides routine obstetric services to a large Australian city, with approximately 4500 deliveries per year. The study was approved by the Human Research Ethics Committees of the Royal Brisbane and Women’s Hospital (HREC/10/QRBW/139) and Queensland University of Technology (1000000558), and conducted in accordance with ethical standards for human research. A consecutive sample was recruited via mail out and/or face-to-face invitation at first clinic visits over a 6 month period beginning in August 2010. All women referred for antenatal care were eligible unless they had insufficient English language skills to complete questionnaires or had pre-existing Type 1 or 2 diabetes. Women provided written informed consent to participate. Ethical approval restrictions prevented the collection of information from women who did not consent to participate.

Data was collected at two time points corresponding to routine maternity visits at approximately 16 and 36 weeks gestation. Women’s weight and height measurements were taken using standard clinical procedures by study staff at the first clinic visit. Self completed questionnaires assessed pre-pregnancy weight, eating and physical activity behaviour, knowledge and demographic information at the first visit. Receipt of health professional advice was assessed at both the first and second visits.

### Measures

#### Weight status

Self reported pre-pregnancy weight and measured height was used to calculate pre-pregnancy Body Mass Index (BMI) which was categorised as: healthy weight (BMI<25 kg/m^2^) and overweight (BMI≥ 25 kg/m^2^) [13,14]. Self reported pre-pregnancy weight is widely used in population studies [15,16] and has been shown to be a reasonable estimate of weight at conception [17].

#### Knowledge

Knowledge regarding the influence of nutrition and physical activity for maternal and offspring health (referred to as pregnancy specific knowledge) was assessed using three items from a previously validated questionnaire [18] with slight modification: “*what a pregnant woman eats during pregnancy has no effect on her health*”, “*what a pregnant woman eats during pregnancy has no effect on the health of her unborn baby*” were derived from a single question in the original validated questionnaire that was considered to be double-barrelled. The third item was “*if a pregnant woman eats a healthy diet there is no need for her to be physically active during pregnancy*” and was unmodified from its original version. Response options were *“true”, “false” and “don’t know”.* Knowledge of fruit and vegetable intake recommendations was assessed using two items previously used with pregnant women [19]: “*how many serves of fruit [vegetables] should a pregnant woman eat for good health”*. A “*don’t know”* option was provided. Definitions of one serve of fruit and one serve of vegetables was provided to assist women to complete the questionnaire. Pregnancy specific knowledge (score 0–3) and knowledge of recommendations (score 0–2) were created by summing the correct items within each index.

Knowledge of recommendations for physical activity in pregnancy was assessed with one item: “*As best you know what is the recommended amount of physical activity for a healthy pregnant woman?”* Four response categories were provided including the correct one of 30 minutes every day [9].

#### Dietary behaviour

The Fat and Fibre Behaviour Questionnaire (FFBQ) [18] is a 20 item questionnaire assessing dietary behaviours (frequency of consumption and use of food items and categories), that influence fat and fibre intake [20]. It provides information on general food patterns rather than specific energy and macronutrient intake. Nine items relate to frequency of consumption of particular high fat or high fibre foods, measured on a five point scale (ranging from 5= Never to 1= 6 or more days per week) [20]. Nine items ask about behaviour relating to cooking, eating or choice of foods such as type of dairy products or bread, measured on a five point scale (ranging from 1= Never, to 5= Always) with a “Not applicable or do not know” option for those who do not eat a particular food or are not aware of specific cooking methods [20]. Two items assessed the number of serves of fruits (1 item) and vegetables (1 item) consumed each day. These two items contribute to the Fibre and total FFBQ scores and are also are valid stand alone measures of fruit and vegetable intake [21].

For each participant an average FFBQ score was calculated across all 20 items and for the Fat (13 items) and Fibre (7 items) subscales. Items associated with unhealthy behaviours were reverse scored. The range for total and subscale scores were 1–5, with higher scores indicating healthier diet quality. Categorical variables were also created to compare the frequency of consumption of specific non-core food groups (outlined in corresponding results table) by combining “less than once a week” or “never” compared to “1-2 times per week” or more. These categories were selected to represent the recommended consumption of non-core foods considering the response options available in the validated questionnaire. Items relating to cooking, eating and choice of foods were dichotomised as “never/rarely” and “sometimes/usually/always”. Serves of fruits and vegetables were compared to recommendations for Australian pregnant women [22].

#### Physical activity

Self reported physical activity (PA) was assessed using the Active Australia Survey items (AAS) [23] which have previously been used in pregnant women. The items assess frequency and duration of walking, moderate and vigorous physical activities. Total number of sessions and minutes of physical activity were each treated as continuous variables. Values greater than 840 minutes were recoded to this value to avoid over-reporting in accordance with recommendations for use of survey items [23]. Categorical variables were also created for sufficient minutes of physical activity (≥150 minutes per week), sufficient sessions (≥ 5 sessions per week) and sufficient activity (≥150 minutes per week + ≥ 5 sessions per week) [23].

#### Support women receive and want

Five items each assessed the frequency of receiving health professional advice on healthy eating and physical activity rated on a five point scale. Results were highly skewed and were dichotomised for the analyses (never/rarely, vs. sometimes/usually/always), to reflected the desired frequency of health professional advice. The specific items were asked at 16 and 36 weeks gestation and are outlined in the corresponding results table.

For the purpose of future intervention and service delivery, at 36 weeks gestation participants were asked if education was available would they have been interested in attending (response yes/no). Those who responded yes were then asked a series of questions regarding their preferred type of education (7 options- written information, individual consultation, group education, combined group and individual education, a website or website with chat forums, a DVD, and a social networking site); when they would have liked to receive this (3 options- when they first found out they were pregnant, at the time of their first antenatal clinic appointment, and specification of another time) and their preferred method of receiving ongoing support or contact (5 options- none, one visit would be enough, telephone, email, face to face/in person, and SMS). Participants were asked to select only one response for each question.

### Statistical analysis

Analyses were performed using Statistical Package for Social Sciences (Version 18, SPSS Inc., Chicago, IL. USA). All data were assessed for normality. All available data were included in analysis. The n value was specified for completeness where values varied. Mean and standard deviation is reported for normally distributed data. Median and interquartile range is reported for skewed data. The criterion for statistical significance was set at the conventional level of *p*<0.05 (two tailed) for all analyses. Differences between groups were assessed using independent sample t tests or Mann Whitney U tests for continuous variables, and chi squared tests for categorical variables.

## Results

Of 1059 eligible women, 664 (63%) consented to participate and provided measured anthropometric data at their first visit and/or a completed questionnaire (n=582, 87% provided both). Mean gestation at recruitment was 16± 2 weeks, mean age was 29 ± 5 years and 60% were first-time mothers. One third (34%) were overweight based on self reported pre-pregnancy weight. Detailed anthroprometic characteristics of the cohort have been reported elsewhere [3]. Table 1 outlines demographic characteristics of healthy and overweight women. Participants were representative of the Queensland obstetric population for age, marital status, ethnicity, parity and anthropometric characteristics [24].

**Table 1:** Participant characteristics at baseline by pre-pregnancy weight status [percentage (count)]

**Characteristic**	**Whole sample (n=582)**	Healthy weight^*^ ^**(n=386)**	**Overweight**^**+^ **^**(n=196)**	**Difference**^**a**^
Age in years mean ± s.d. (range)	29.9 ± 5.1 (17–45)	29.9 ± 5.2 (17–45)	30.0± 5.1 (18–42)	p=0.809
Parity Nulliparous	60.6 (351)	63.3 (243)	55.4 (108)	p=0.072
Marital Status Married	62.3 (361)	62.1 (239)	62.9 (122)	p=0.484
Defacto	32.2 (187)	31.9 (123)	33.0 (64)	
Single	5.3 (21)	6.0 (23)	4.1 (8)	
Education Year 10 or less	7.6 (44)	7.1 (27)	8.2 (16)	p=0.003
Year 12 or equivalent	14.6 (85)	14.4 (55)	14.9 (29)	
Vocational training^‡^	33.0 (192)	29.0 (111)	41.0 (80)	
University or higher university degree	44.8 (261)	49.6 (190)	35.9 (70)	
Income^b^ Low income $50 000 or less	23 (110)	20 (66)	26 (44)	p=0.089
Middle income $50001 to 100000	50 (247)	48 (158)	52 (89)	
High income >$100000	28 (142)	31 (103)	23 (39)	
Birth country Australia	70 (404)	65 (251)	79 (153)	p=0.001
Language other than English at home	13.8 (80)	16.9 (65)	7.7 (15)	p=0.003

^*^Healthy weight= pre-pregnancy Body Mass Index <25 kg/m^2^; ^+^Overweight= pre-pregnancy Body Mass Index ≥25 kg/m^2^; ^**^**^measured height and self reported pre-pregnancy weight; ^a^test for significance chi squared; ^b^n=499 total (healthy weight n=247, overweight n=147); ^‡^trade, apprenticeship, certificate or diploma.

### Knowledge

Four percent of participants achieved the maximum knowledge score for both pregnancy specific nutrition knowledge and knowledge of recommendations. The majority of women (92%) correctly answered the three pregnancy specific nutrition questions (outlined in the knowledge section in methods) (mean = 2.9 ± 0.4). Eight percent and 36% of study participants could correctly report the recommended daily number of fruit and vegetable serves respectively. Sixty nine percent (n=378) correctly identified 30 minutes a day as the recommended amount of physical activity for a pregnant woman. There were no differences between healthy and overweight women on any knowledge measures.

### Dietary behaviour

The mean FFBQ score was 3.2 ± 0.5 (range 1–5, higher score indicates better diet quality). Total FFBQ score, or Fat subscale scores were not different between healthy weight and overweight groups (Table 2). Only 4% of participants ate the recommended 5 serves/day of vegetables with no group differences. More healthy weight participants achieved the recommended fruit serves (4 serves/day) compared to overweight women (8% vs 4%, *χ*^2^=3.86, *df* 1, p=0.05).

**Table 2:** 16 week dietary behaviour measures for the whole sample and by pre-pregnancy weight status [Mean ± s.d or percentage (count)]

	**Whole sample (n=575)**	Healthy weight^*^ ^**(n=382)**	**Overweight**^**+^ **^**(n=193)**	**Difference**
Total FFBQ score^a^	3.2 ± 0.5	3.2 ± 0.5	3.2 ± 0.5	0.431
FFBQ fat subscale score	3.4 ± 0.5	3.4 ± 0.6	3.4 ± 0.5	0.565
FFBQ fibre subscale score	2.8 ± 0.6	2.9 ± 0.6	2.7 ± 0.6	0.009
Serves of fruit/day (4/day recommended)	2.0 ± 1.0	2.1 ± 1.0	1.8 ± 0.9	0.001
Serves of vegetables/day (5/day recommended)	2.3 ± 1.2	2.3 ± 1.2	2.3 ± 1.1	0.896
Usually/Always choose reduced fat milk	57 (329)	56 (215)	59 (114)	0.547
Usually/Always choose reduced fat cream including ice-cream	56 (322)	54 (206)	60 (116)	0.151
Usually/Always choose reduced fat cheeses	37 (214)	35 (133)	42 (81)	0.090
Take-away consumed once/week or more	29 (168)	25 (95)	37 (72)	0.002
Soft drink/cordial consumed once/week or more	47 (274)	43 (165)	55 (108)	0.005
High fat savoury biscuits consumed once/week or more	27 (155)	24 (91)	32 (63)	0.025

^*^Healthy weight= pre-pregnancy Body Mass Index <25 kg/m^2^; ^+^Overweight= pre-pregnancy Body Mass Index ≥25 kg/m^2^; ^^^measured height and self reported pre-pregnancy weight; FFBQ= Fat and Fibre Behaviour Questionnaire [18], ^a^score scale ranges from 1 lowest to 5 highest quality of intake.

Table 2 outlines the proportion of women usually/always choosing reduced fat dairy products. There were no differences between weight status groups. The consumption of take-away, softdrink/cordial and high fat savoury biscuits once a week or more was higher in overweight compared to healthy weight women (Table 2). There was no difference between groups reporting consumption once a week or more of chocolate and lollies; pastries, cakes and biscuits; potato crisps, corn chips or salted nuts; and potato chips, french fries or wedges.

### Physical activity

Continuous physical activity measures were positively skewed. Details of the number of sessions, minutes for each activity and totals are presented in Table 3. Walking was the predominant activity reported by study participants. Forty four percent of participants met guidelines for both session and minutes of physical activity. There was no difference between healthy and overweight participants achieving sufficient total sessions, minutes of activity or sessions and minutes combined (Table 3).

**Table 3:** 16 week physical activity measures per week for the whole sample and by pre-pregnancy weight status [Median (interquartile range) or percentage (count)]

	**Whole sample (n=575)**	Healthy weight^*^^**(n=382)**	**Overweight**^**+^ **^**(n=193)**	**Difference**
Sessions of walking	4 (2–6)	4 (2–6)	4 (2–5)	0.066^b^
Minutes of walking	120 (50–180)	120 (60–206)	120 (45–180)	0.214^b^
Sessions of moderate activity	0 (0–1)	0 (0–1)	0 (0–1)	0.968^b^
Minutes of moderate activity	0 (0–60)	0 (0–60)	0 (0–45)	0.734^b^
Sessions of vigorous physical activity	0 (0–1)	0 (0–1)	0 (0–0)	0.026^b^
Minutes of vigorous physical activity	0 (0–25)	0 (0–30)	0 (0–0)	0.026^b^
Total sessions of physical activity	5 (3–8)	5 (3–8)	5 (3–7)	0.044^b^
Total minutes of physical activity	150 (60–285)	150 (72–300)	150 (60–247)	0.155^b^
Sufficient sessions physical activity	58 (328)	59 (219)	55 (107)	0.376^c^
Sufficient minutes of physical activity	51 (291)	51(192)	51 (97)	0.925^c^
Sufficient sessions and minutes of physical activity	44 (245)	44 (160)	43 (83)	0.934^c^

^*^Healthy weight, pre-pregnancy Body Mass Index <25 kg/m^2^; ^+^Overweight, pre-pregnancy Body Mass Index ≥25 kg/m^2^; ^a^measured height and self reported pre-pregnancy weight; ^b^independent samples Mann–Whitney *U* Test; ^c^chi squared test.

### What support women are receiving

The proportion of participants sometimes/usually/always being provided with health professional advice relating to healthy eating and physical activity in pregnancy at 16 and 36 weeks gestation is outlined in Table 4. There was no difference between the proportions for healthy and overweight women (data not presented). There was little change in the reported proportions at 36 weeks gestation.

**Table 4:** Reported proportion of “sometimes-always” being provided with health professional advice relating to healthy eating and physical early and later in pregnancy [percentage (count)]

**The health care professionals who have cared for me since I became pregnant....**	**16 weeks gestation “sometimes-always” (n=575)**	**36 weeks gestation “sometimes-always” (n=492)**
Ask me about the foods I eat	43 (247)	39 (191)
Encourage me to eat healthy foods	64 (373)	58 (287)
Give advice about the amount of food to eat	29 (169)	21 (104)
Give advice about how to plan and prepare healthy food	16 (95)	13 (65)
Ask me about the physical activity I do	39 (226)	42 (206)
Encourage me to be physically active	47 (273)	50 (243)
Advise me to limit the amount of activity I do	23 (135)	18 (89)
Criticise me for not doing enough physical activity	4 (21)	3 (14)
Offer advice about how to include physical activity in my day	23 (135)	21 (101)

### What support women want

Eighty percent of participants stated they would have been interested in attending education about nutrition, physical activity and weight control if it was available with no difference in responses between healthy and overweight women. Of those women wanting education, a social networking site (6%), DVD (10%) and group sessions (11%) were the least popular means of education. Written information (40%), individual education (26%) and a combination of group and individual sessions (25%) were most frequently selected preferred methods of education. This information needs to be interpreted with caution as some women selected only their most preferred option (one option) where as others selected multiple methods. Fifty-five percent of women wanted information when they first found out they were pregnant, 35% when they first came to clinic and 10% at another time. Those who reported “at another time” provided comments most relating to wanting information at multiple times through the pregnancy. Preferred method of ongoing contact was email (43%), face to face (36%), telephone (20%) and text message (6%).

## Discussion

This is one of the first studies to simultaneously examine diet and physical activity knowledge, lifestyle behaviour according to pre-pregnancy weight status, and health professional advice received. While pregnancy specific nutrition knowledge about the influence of nutrition on maternal and offspring health was good, knowledge regarding recommendations for fruit and vegetable consumption was poor. Less than one in ten women met key dietary recommendations. Only 44% achieved sufficient physical activity recommendations. While half to two thirds of women reported health professionals encouraged physical activity and healthy eating respectively, specific advice and support to achieve these healthy lifestyle behaviours was limited.

Knowledge is considered a core determinant for behaviour change [10] and nutrition knowledge has been independently associated with intake of fruit and vegetables in men and women [25]. New Beginnings study participants correctly identified that diet during pregnancy affects maternal and child health and that it is important to eat well and be physically active (pregnancy specific knowledge). However women were knew less about the practical application of this knowledge such as the recommendations for the amount of fruit and vegetables to consume. Knowledge deficits were similar for healthy weight and overweight pregnant women. Women cannot be expected meet recommendations without adequate knowledge of what should be achieved; however this alone may not result in appropriate behaviour. Once women have adequate knowledge, supportive behaviour change strategies can be utilised to assist women achieve recommendations.

The nutrition and physical activity results of the New Beginnings study are consistent with results from other Australian studies. New Beginnings participants reported an average consumption of fruit and vegetables of two serves each per day. This is the same as reported in two Australian studies using similar methods as the current study [6,7]. Very few women appear to be achieving sufficient fruit (4 serves per day) and vegetables (5 serves per day) with less than ten percent of both New Beginnings participants and another Australian population achieving these [6]. Only two in five New Beginnings participants were sufficiently active, a similar proportion to another study using the same measures later in pregnancy, where one-third were sufficiently active [6]. Identifying successful strategies to support women achieve lifestyle recommendations during pregnancy are needed.

Broadly our results suggest pre-pregnancy weight status had little impact on those achieving recommendations with no differences between healthy and overweight women achieving serves of vegetables, and sufficient physical activity; overall dietary quality score and fat subscale score. However overweight women had a lower fruit intake, participated in less vigorous physical activity and were more likely to consume some energy-dense and nutrient poor foods once a week or more. This suggests women commencing pregnancy overweight may need targeted advice to address specific behaviour rather than general advice. Vulnerable groups, such as the economically disadvantaged and those socially and geographically isolated, have a greater burden of chronic disease compared to the remainder of the population [26]. Higher pre-pregnancy BMIs have been correlated with social and economic problems, with more unemployment and burdensome jobs [27]. It is likely that higher maternal BMI is associated with lower socioeconomic standing, and general advice may be insufficient to overcome specific barriers to engaging in healthy lifestyles.

A potential limitation in detecting group differences for dietary quality was the limited range of items in the tool and the categorisation of pre-pregnancy BMI. Previous studies using 90–120 item tools have identified a negative association between pre-pregnancy BMI and dietary quality [28,29]. Laraia et al. (2007) [28] found differences between obese women and healthy and underweight women for total dietary quality, but pre-obese women were not different from any weight category [28]. It is possible that combining pre-obese and obese women in the current sample ameliorated differences between healthy weight and overweight women. Further our 20 item tool may not have enough diversity in dietary items to detect differences in overall diet quality between weight status categories.

Health professionals providing antenatal care have an important role in advising and supporting women achieve recommendations for health behaviour in pregnancy. However in the current study support and advice beyond encouragement was limited. This is consistent with studies examining antenatal care provider practices in the context of weight gain and obesity [11,30-32]. Despite health care providers acknowledging the importance of nutrition and physical activity [11,30,31], a lack of knowledge and education has been reported [30,31,33-36]. This knowledge gap needs to be addressed if health care providers are to have the confidence to provide appropriate advice and support women achieve recommendations during pregnancy.

In contrast to the lack of advice women reported receiving, four out of five women were interested in receiving education; they wanted this individualised and preferred face to face contact. These results are consistent with previous studies [37,38], where women wanted a tailored approach to care [38]. These results highlight the disparity between what women want and what current services may be providing. Targeting intervention delivery to meet the needs of women is important to ensure engagement with services; however this needs to be balanced with the ability of health services to deliver.

The New Beginnings study was a large consecutive sample that was representative of the hospital and state population from which it was recruited for age, ethnicity, marital status and anthropometric characteristics. Ethical approval prevented data collection about non-consenters to the study. It is possible our study population is socioeconomically advantaged compared with the rest of the state, evidence by a high proportion of university educated women. This is likely to underestimate the magnitude of the knowledge and behavioural deficits represented here. The simultaneous assessment of knowledge, behaviour and advice received adds strength to the findings reported. However these results need to be considered in the context of self reported measures. Dietary intake and physical activity behaviour may be prone to reporting bias in a socially desirable direction, with overweight women potentially more likely to do this. While objective measures may have strengthened findings, the tools selected were valid and reliable [20,23]. Health care providers views of advice provision were not assessed as the women’s recall of advice will ultimately guide behaviour [39]. It is possible that women underreported the health care advice received, however this would not account for the very low level of advice observed in these study results.

## Conclusions

These results suggest that basic recommendations for a healthy pregnancy were not being met by women in our antenatal service in relation to eating and physical activity. Overweight women appear less likely to do so for some but not all recommendations. This is perhaps not surprising in the context of relatively poor knowledge and limited health care professional assessment or advice. There are opportunities to improve the health care services pregnant women received to improve knowledge and behaviours related to achieving a healthy lifestyle, and indeed it appears pregnant women want this.

## Competing interests

The authors declare they have no competing interests associated with this manuscript.

## Authors’ contributions

SdeJ conceived and designed the study with support from JN, LC and LD. SdeJ collected the data and performed the analysis. All authors contributed to the interpretation of results, drafting of the manuscript, and approved the final manuscript for submission.

## Pre-publication history

The pre-publication history for this paper can be accessed here:

http://www.biomedcentral.com/1471-2393/13/115/prepub
